# Wishful thinking versus operational commitment: is the international guidance on priority sexual and reproductive health interventions in humanitarian settings becoming unrealistic?

**DOI:** 10.1186/s13031-018-0157-x

**Published:** 2018-05-29

**Authors:** Nguyen Toan Tran, Catrin Schulte-Hillen

**Affiliations:** 1 Australian Centre for Public and Population Health Research, Faculty of Health, University of Technology, PO Box 123, Sydney, NSW 2007 Australia; 2 Institute of Demography and Socioeconomics (IDESO), University of Geneva, 40 bd Pont d’Arve, 1211 Genève 4, Switzerland; 3 Médecins sans Frontières, Rue de Lausanne 78, 1202 Genève, Switzerland

## Abstract

Twenty-one years ago, a global consortium of like-minded institutions designed the landmark Minimum Initial Service Package (MISP) for sexual and reproductive health (SRH) to guide national and international humanitarian first responders in preventing morbidity and mortality at the onset of chaos, destruction, and high insecurity caused by disasters or conflicts. Since then, the MISP has undergone limited change and has become an international reference in humanitarian response. This article discusses our perspectives regarding the 2018 changes to the MISP that have created division among humanitarian field practitioners, academics, advocates, and development agencies. With more than 50 pages, the new MISP chapter dilutes key guidance and messages on the most life-saving activities, leaving actors with excessive room for interpretation as to which priority activities need to be first implemented. Consequently, non-life-saving interventions may take precedence over essential ones. Insecurity, scarce human and financial resources, logistics constrains, and other limitations imposed by field reality at the onset of a crisis must be considered. We strongly recommend that an institution with the mandate, legitimacy, and technical expertise in the review of guidelines reexamines the 2018 edition of the MISP. We urge experienced first-line responders, national actors, and relevant agencies to join efforts to ensure that the MISP remains focused on a very limited set of essential activities and supplies that are pragmatic, field-oriented, and, most importantly, immediately life-saving for people in need.

## Background

In the face of the massive scale of sexual violence during the Rwandan genocide and its aftermath, and following the 1994 International Conference on Population and Development (ICPD) in Cairo, which enshrined access to sexual and reproductive health (SRH) for refugees and internally displaced populations, the Inter-Agency Symposium on Reproductive Health in Refugee Situations gathered UN agencies, NGOs, donors, and academic institutions in Geneva in 1995. This consortium established the Inter-Agency Working Group (IAWG) for Reproductive Health in Crises. In 1996, the IAWG designed the Minimum Initial Service Package (MISP) for SRH to guide first responders in emergencies in preventing SRH-related morbidity and mortality. The MISP pursues five objectives, each objective with its specific activities, to be implemented as a priority at the onset of a humanitarian response to an emergency. Two objectives focus on coordination and planning (ensuring the health sector/cluster identifies an organization to lead implementation of the MISP; planning for comprehensive SRH services, integrated into primary health care as soon as possible) and three objectives address the provision of SRH services that aim to save lives or alleviate diseases and suffering during the initial phase of an emergency (prevent sexual violence and respond to the needs of survivors; prevent the transmission of and reduce morbidity and mortality due to HIV and other sexually transmitted infections; prevent excess maternal and newborn morbidity and mortality).

The MISP is outlined in the Inter-Agency Field Manual (IAFM) on Reproductive Health in Humanitarian Settings, which was first published in 1999. Over the past 20 years, the MISP has become a minimum standard in humanitarian response and its objectives and related activities underwent technical updates and some modification and wording change aimed at improving its clarity during the IAFM revision in 2010.

## An inadequate revision process

The IAWG undertook another revision of the IAFM over the 2016–2017 period, involving “a deliberate collaborative process that included hundreds of individuals from dozens of agencies and organizations working in humanitarian settings at global, regional, and local levels. The updates to the IAFM…represent the consensus positions of a wide cross-section of agencies working on sexual and reproductive health in the humanitarian sector” [1].

We fully acknowledge the efforts made to raise and debate the new options for the MISP in the IAFM revision. The adopted revision process was very lengthy because it aimed to be inclusive of the growing number of IAWG partners (many of them North American) and the diversity of views, including those of advocates, academics, development institutions, and a minority of humanitarian agencies. It did not favor the participation and the voices of the more pragmatic and field-oriented humanitarian actors – there were no national actors involved in humanitarian preparedness and response in their countries. When it comes to setting standards, it is critical to take stock of more than 20 years of experience in MISP implementation. Most of the proposed changes (see examples below) were pushed through by IAWG sub-working groups with their own single-issue priorities on abortion, contraception or rights advocacy, without much consideration of MISP implementation challenges and its objectives as a whole.

The responsibility of altering the MISP should be collaborative but not dependent on a democratic voting process among IAWG members that creates a bias towards the opinion of advocacy, rights-based, academic or other institutions that have no experience or first-line responsibilities in the acute phase of a humanitarian response: in the end, they are not the agencies setting up services nor is their staff living with the day-to-day trade-offs between limited resources, protecting rights, and deciding what can be done amidst chaos, insecurity, and political uncertainty. Therefore, we strongly recommend that the currently finalized MISP guidance be reexamined by an institution with the mandate, legitimacy, and technical excellence in guideline review. The process should not only be based on scientific evidence, but also on acceptability and pragmatic considerations in the field. It must therefore involve relevant national stakeholders along with  international actors. To further legitimize the revision process vis-à-vis national stakeholders and humanitarian partners, the World Health Organization (WHO) must be engaged, as well as the United Nations High Commissioner for Refugees (UNHCR) and the United Nations Population Fund (UNFPA). UNHCR and UNFPA directly coordinate with local authorities and partners to ensure that the MISP is implemented in humanitarian settings. The final decision on the 2018 and future revisions of the MISP guidance must be the responsibility of experts and experienced field implementers who have direct accountability for emergency medical interventions. Therefore, we feel compelled to reiterate our concerns vis-à-vis the revision process and some of the changes in the MISP, which is the cornerstone of the IAFM, as they may fail to ensure the most basic medical care required by women and girls at the onset of an emergency.

## “Mist in the MISP”

During its 16th annual meeting which took place in March 2016, the IAWG, which carried out a global evaluation of its work since 2004, took stock of advances in, and remaining challenges for SRH in humanitarian settings [2]: as a result of the IAWG partners’ efforts, the MISP had become a well-known and widely applied reference for action in humanitarian settings; an increased number of institutions provided MISP-related and comprehensive SRH services; the availability of abortion-related services and the provision of long-acting and reversible contraceptive methods were found to be lagging; only a third of institutions working in the field reported appointing an SRH coordinator; and planning for comprehensive SRH remained often misunderstood or challenging to implement. Given the diverse nature of humanitarian crises, some IAWG members have argued that the MISP is not adapted to different settings or to current emergencies. Other members have advocated for stronger emphasis on services that were found to be weak in the 2014 Global Evaluation, such as contraception or abortion care. This led to different ideas and discussions to complement the MISP with additional objectives and activities to form a “MISP+” set of interventions. In 2016, we pointed to the fact that there was still “mist in the MISP” and how proposed changes to the MISP hinge on the lack of understanding of (i) the purpose of the MISP (priority life-saving SRH interventions that can be implemented during the chaotic first days of a humanitarian response without an in-depth SRH needs assessment), (ii) the often very limited operational and technical capacity of national and international actors, and (iii) two crucial, interconnected MISP objectives: (1) planning for comprehensive SRH, which in turns depends on (2) the (too-rarely-done) systematic appointment of an effective SRH coordinator whose role it is to manage MISP implementation and the transition towards comprehensive SRH once the situation has stabilized. Therefore, we recommended to leave the MISP guidance as such intact and to focus efforts on developing the capacity of IAWG institutions in terms of operational and technical capacity and on the management and leadership objectives of the MISP: SRH coordination and planning for comprehensive SRH. Nevertheless, the IAFM revision process adopted changes that we believe will contribute to compromise the implementation of immediately life-saving MISP services by diverting resources and attention to other activities. For example:

First, addition of a new objective and related activities on contraception: (Objective:) “prevent unintended pregnancies” by (Activity 1:) “ensuring availability of a range of long-acting reversible and short-acting contraceptive methods (including male and female condoms and emergency contraception) at primary health care facilities to meet demand”; by (Activity 2:) “providing information, including information, education, and communication (IEC) materials, and, as soon as possible, ensuring contraceptive counseling that emphasizes informed choice, effectiveness, and supports client privacy and confidentiality”; and by (Activity 3:) “ensuring the community is aware of the availability of contraceptives for women, adolescents, and men.” Activity 3 has two sub-activities: (Sub-activity 3.1:) “Ensure the community is aware of where and how to seek access to contraception, including unmarried and adolescent community members. Information should be communicated in multiple formats and languages to ensure accessibility (e.g., Braille, sign language, pictograms and pictures)”; (Sub-activity 3.2:) “Engage community leaders to disseminate information about availability of contraceptive services.” In the 2010 version of the MISP, the recommended activity was succinctly put: “provide contraceptives, such as condoms, pills, injectables, and intra-uterine devices (IUD) to meet demand” and commonly appended to the objective “prevent excess maternal and neonatal morbidity and mortality.” Second, to the objectives, a “note”, which is actually an additional priority, is added: “It is also important to ensure that safe abortion care is available, to the full extent of the law, in health centers and hospital facilities.” In the original version of the MISP, uterine evacuation is embedded as one of the signal functions needed to “ensure the availability of Emergency Obstetrics and Newborn Care (EmONC).”

## Compromising the implementation of the MISP

### The MISP must remain field-oriented and pragmatic

The MISP is designed to be implemented amid chaos and high insecurity when the health system has been severely impaired, including staff, logistics, infrastructure, and health information systems. In such a setting, there is no time for capacity development of staff, no time for in-depth needs assessment, no time for public health campaigns, comprehensive services, and polished quality of care, all of which are part of the planning for context-appropriate comprehensive services once the situation has stabilized. Therefore, the MISP must remain field-oriented and pragmatic. With more than 50 pages long, the new IAFM chapter on the MISP dilutes key guidance and messages regarding the most essential life-saving activities, with certain activities containing many details (e.g., prescribing emergency contraceptive pills, providing newborn health care), while others have few, such as emergency obstetric care – the most life-saving maternal and newborn health (MNH) interventions. This leaves actors with excessive room for interpretation as to which priority activities should be implemented, supported, coordinated and funded. Consequently, activities that are not immediately life-saving may take precedence over essential ones. Two examples:

First, there should not be a sixth objective - preventing unintended pregnancies. Although currently a global focus of FP2020 and other planetary health initiatives, preventing unintended pregnancies through contraception remains a cornerstone of MNH along with EmONC and should therefore be subsumed under the already existing MNH objective. There is no time, especially when EmONC services are not yet set up, to start comprehensive contraceptive programming at the onset of an emergency, “to engage community leaders…” or design and provide IEC materials “in multiple formats and languages to ensure accessibility (e.g., Braille,…).” In addition, “counseling that emphasizes informed choice, effectiveness, and supports client privacy and confidentiality” are crosscutting rights-based quality of care musts, which have been advocated for by WHO but are not restricted to contraceptive services: it applies to all SRH and health services [3]. Therefore, it should be moved out from these activities and integrated into the overall quality of care objectives ensured by SRH providers, managers, and coordinators.

Second, all affected populations, including subgroups, such as people living with disability, adolescents, older people, sex workers, or members of the lesbian, gay, bisexual, transgender, queer/questioning, intersex, and asexual community, among others, should have access to life-saving SRH services. Non-discrimination, safety, respect, and confidentiality are already embedded in the overarching values necessary to the MISP implementation. The new MISP chapter expands on subgroups and proposes targeted activities, such as engagement with these communities and training providers on their specific needs. Such activities, to be meaningful and of high quality, cannot be done in a “touch-and-go” fashion amid chaos and insecurity and require in-depth needs assessment and planning with the populations of concern. Therefore, this should not be part of MISP, but needs to be an essential part of planning for comprehensive programming and emergency preparedness plans.

### The implementation of the MISP is constrained by logistics

At the onset of disasters, like the tsunami in 2004, the earthquake in Haiti in 2011 or in Nepal in 2015, or in war-torn situations such as Yemen or South Sudan, ensuring availability of life-saving supplies presents a colossal challenge. The choice of supplies that need to be financed, transported - and stored - should be informed by the MISP activities and be obvious to field actors. Within the supply chain, adding a non-essential supply is a trade-off for another essential one. The MISP should therefore remain focused on a very limited set of essential activities and related supplies for the initial response.

The proposition to add lubricants and female condoms to the activity to ensure condom availability – eventually removed from the final version - exemplifies how some of the proposed recommendations departed from logistics and field realities. Female condoms should only be made available during the emergency response if pre-existing demand is known, otherwise they will go to waste. WHO recommends lubricants for MSM and sex worker programs, however with different specificities for anal sex and vaginal sex [4]. In addition, some lubricants may increase the risk of epithelial damage and must be carefully chosen [4]. Finally, the condoms in the inter-agency SRH kits that support the implementation of the MISP are already lubricated and including additional lubricants will not only increase significantly the price of shipping by air but will be done at the expense of other life-saving supplies – such trade-off choices should not exist in an emergency. Likewise, a focus on making long-acting reversible contraceptives systematically available within a range of methods in all settings should take into consideration the risk of IUDs or implants going to waste if they were not known and used by the affected communities prior to the crisis. In addition, women who receive IUDs and implants in acute humanitarian context may not have access during their forced displacement to counseling on side-effects and contraceptive removal services. Such methods should be introduced as part of the design of quality comprehensive SRH services.

### Human rights in the face of insecurity and realpolitik

The right to medical care is a human right which applies to access to safe abortion care (SAC). Institutional and staff resistance and lack of capacity to provide SAC are widespread in stable contexts and even in contexts where it is legal. Why aid organizations are not providing SAC to avert one of the main causes of maternal deaths and suffering has been debated at length. This includes insights into the vast field experience of MSF and the enormous amount of investment and training needed to just even scratch the surface of the issue in field operations, among staff, and within the affected communities [5], and was demonstrated by the presentations on SAC programs during the recent 2017 IAWG annual meeting (none presented a SAC program during an acute phase response and all of the presenters agreed that this would be very difficult). Amid chaos and insecurity, openly focusing on such a sensitive issue may put field staff, patients, and operations at risk. Making SAC visibly upfront in the MISP – even as a “note” and not as an “objective” -- is commendable but will render the work of implementing agencies problematic. Host governments and local authorities, to avoid conflict with national legislations, may be reluctant to conclude working agreements with IAWG-signatory organizations, thus potentially delaying the operational set-up and paralyzing the implementation of other MISP activities and assistance to people in need because of this single issue. For example, in the aftermath of the 2008 cyclone Nargis in Myanmar, local authorities forbid the custom clearance of inter-agency SRH kits because of kit 8, which was then named “management of abortion complications.” Kit 8, which contains manual vacuum aspiration and misoprostol, is necessary to perform uterine evacuation (one of the seven EmONC signal functions). To avoid repetition in the future, kit 8 is now re-named without the “abortion” word: to “manage the complications of *miscarriages*.” This practical example illustrates how the crafting of the MISP is best informed by field practice and reality – as well as realpolitik.

## Conclusions

The success of the IAWG is largely due to its openness and inclusiveness towards a large and growing range of institutions working on SRH. Many of these institutions pursue diverse agendas, including advocacy, human rights, development, and research. Using the MISP as an advocacy vehicle to defend with fervor and pride comprehensive contraception services and SAC has overshadowed the unmet need for early transition from MISP to comprehensive SRH services. This has raised doubts to what extent the various actors have a shared understanding of impartiality in humanitarian crisis settings. The revision process of an operational guidance document, which may be enriched by different viewpoints, needs to respond first and foremost to the challenges encountered by field actors, and be led by an institution that has the mandate, expertise, and legitimacy to do so. In addition to coordination and early planning for context-appropriate quality comprehensive SRH services, the MISP needs to remain a relevant must among donors and humanitarian responders in times of scarce resources and limited capacity. It must focus on a very limited set of essential activities and supplies that are pragmatic, field-oriented, and, most importantly, immediately life-saving for people in need.

## Abbreviations

EmONC: Emergency Obstetrics and Newborn Care
IAFM: Inter-Agency Field Manual on Reproductive Health in Humanitarian Settings
IAWG: Inter-Agency Working Group for Reproductive Health in Crises
IEC: Information, education, and communication
IUD: Intra-uterine device
MISP: Minimum Initial Service Package for sexual and reproductive health in crises
MNH: Maternal and newborn health
SAC: Safe abortion care
SRH: Sexual and reproductive health
UNFPA: United Nations Population Fund
UNHCR: United Nations High Commissioner for Refugees
WHO: World Health Organization
